# Adult-Child Caregivers’ Family Communication Experiences after an Older Parent’s Blood Cancer Diagnosis: A Survey Exploring Their Openness, Avoidance, and Social Support

**DOI:** 10.3390/cancers15123177

**Published:** 2023-06-14

**Authors:** Kevin B. Wright, Carma L. Bylund, Taylor S. Vasquez, M. Devyn Mullis, Maria Sae-Hau, Elisa S. Weiss, Diliara Bagautdinova, Carla L. Fisher

**Affiliations:** 1 Department of Communication, College of Humanities and Social Sciences, George Mason University, Fairfax, VA 22030, USA; 2 Department of Health Outcomes and Biomedical Informatics, College of Medicine, University of Florida, Gainesville, FL 32611, USA; carma.bylund@ufl.edu (C.L.B.); mullis.md@ufl.edu (M.D.M.); carlalfisher@ufl.edu (C.L.F.); 3 Department of Advertising, College of Journalism and Communications, University of Florida, Gainesville, FL 32611, USA; tsthelander@ufl.edu (T.S.V.); dbagautdinova@ufl.edu (D.B.); 4 The Leukemia & Lymphoma Society, Rye Brook, NY 10573, USA; maria.sae-hau@lls.org (M.S.-H.); elisa.weiss@lls.org (E.S.W.)

**Keywords:** adult child, aging parent, blood cancer, caregiving, family communication, social support

## Abstract

**Simple Summary:**

Adult children in midlife often become their aging parent’s caregiver after a blood cancer diagnosis. Navigating this role reversal during midlife is an additional challenge. Adult children juggle multiple roles (as spouse, parent, and professional). Blood cancer caregiving also involves unpredictable challenges. These challenges can make communicating even more complex. We explored what adult-child caregivers do to enhance their family’s communication and what they struggle with when talking to diagnosed parents. A total of 124 adult-child caregivers participated in an online survey. Results showed that caregivers perceive that family communication is enhanced when they communicate openly and frequently. They use technology to maintain contact, facilitate connectedness, share information, and encourage involvement. Diagnosed parents and their adult children struggle with being open. Yet, when they communicate more openly about cancer with family, they have more support. Interventions could help caregivers take the lead in facilitating openness and support after their parent’s blood cancer diagnosis.

**Abstract:**

Adult-child caregivers of an aging parent living with a blood cancer describe struggling to communicate with one another and within the family system. They may avoid critical care conversations, which may impede care and their ability to receive social support. We examined what approaches adult-child caregivers of a parent diagnosed with a blood cancer use to enhance their family communication, the topics they find most challenging to discuss, and the roles of openness and support. We used qualitative and quantitative approaches to analyze data from a larger online survey study. In partnership with the Leukemia & Lymphoma Society, we recruited 121 adult-child caregivers. Responses to one open-ended item were analyzed to capture strategies used to enhance communication with their parent and family. They reported utilizing digital communication modalities, prioritizing frequent communication, engaging in openness, establishing boundaries, kinkeeping, and enacting support. Within the quantitative data, we further explored two of these themes (openness and support) and their relationships to other variables using *t*-tests and regression analysis. Adult-child caregivers and diagnosed parents avoid talking about mortality and negative feelings. Openness in the family about cancer was linked to caregivers’ perceptions of receiving social support. Findings demonstrate that cultivating openness between midlife adult children and diagnosed parents may enhance opportunities to receive support.

## 1. Introduction

Given that most hematological cancers are diagnosed in the sixth through to the ninth decade of life, midlife adult children are often involved in providing care to a diagnosed parent [1]. Although caregiving involves both benefits and challenges, midlife adult children are at a high risk for negative experiences [2]. Midlife adults juggle multiple roles (as spouse, parent, and professional) [3,4], while managing the many needs of an aging parent with hematological cancer. Moreover, blood cancer caregiving can involve unpredictable challenges, as the cancer and its treatments affect the immune system. Depending on the parent’s blood cancer subtype, such challenges may include multiple emergency department visits, an urgent need for treatment, extended hospitalization, risk of infection, lengthy or indefinite treatment, costly medications, side effects that can be serious or make daily living very difficult, and frequent outpatient visits [5,6,7,8,9,10]. Collectively, cancer caregiving impacts adult-child caregivers’ mental, physical, and relational well-being and increases their socio-emotional burden due to childcare and employment responsibilities [11]. Cancer caregiving experts have called for the development of more supportive resources that help family caregivers such as adult children develop skills central to their ability to provide their parent care and to mitigate adverse caregiving effects [12].

Talking about cancer and caregiving needs during challenging transitions (as well as during day-to-day care across the disease trajectory) is central to family caregivers’ ability to cope, manage distress, fulfill caregiving needs, and promote better health outcomes for themselves, the patient, and family members [13,14,15]. Caregivers may experience difficulty finding the right words [16], fear saying the wrong thing [17], or be met with resistance when communicating with family. Families also do not always communicate openly about health (i.e., are not always willing to communicate) [18,19,20] and may avoid cancer-related topics [21,22]. Cancer-related topic avoidance (the attempt to prevent or terminate the discussion of a particular subject [23]) is common within parent–child caregiver–patient relationships, and while sometimes beneficial (e.g., evading embarrassment), avoidance contributes to poor mental health outcomes for both patients and caregivers [22]. Hiding concerns or not having someone to confide in is linked to distress including more depression and anxiety [24]. In contrast, when families communicate openly, patients report better physical and relational health [25].

However, merely asking an adult-child caregiver to communicate “openly” with their diagnosed parent ignores the complexities of why families struggle with openness or how challenges vary based on contextual factors such as the particular relationship (spouses versus parent–child), illness, culture, and family environment [26]. Resources for caregivers should be tailored to better serve their distinct needs [26,27], which includes helping them learn communication skills related to open and avoidant behavior [13]. A systematic review suggested that caregiver interventions that teach communication skills may decrease burden and enhance quality of life [28]. To identify adult-child blood cancer caregivers’ needs for communication skill development, we must understand the nature of family communication (including openness/avoidance) after a parent’s diagnosis.

### The Challenges of Talking about Cancer: Openness and Avoidance

Families’ willingness and motivations for talking (or not) about cancer vary [29,30,31]. For instance, diagnosed parents or adult children may avoid cancer topics because they feel that the disease is a private issue [25,32,33]. For instance, older generations in the U.S. were typically socialized to keep health issues private, whereas younger generations experienced less privacy boundaries [25]. Avoiding cancer discussions is also sometimes done to maintain a sense of hope or normalcy [21,33]. Patients and their families have reported avoiding excessive talk about cancer to maintain well-balanced lives, focus on enjoyable topics during what may be a limited remaining lifespan, or maintain a hopeful attitude [13,33,34]. In addition, certain cancer-related topics may pose specific challenges. Patients report hesitancy when it comes to sharing distressing feelings (e.g., anger) [35], believing that such disclosures could exacerbate negative affect [25,33,36]. Additionally, midlife children of diagnosed parents describe challenges with sharing information about the diagnosis, decisions, and mortality [29]. They also avoid discussing uncertainty about the future (e.g., parent’s prognosis) and are more open about non-emotional topics such as treatment logistics [25,33,37].

Family social support is also associated with cancer-related topic avoidance [38,39]. Various types of social support may be impeded if families avoid cancer topics [40], whereas sharing cancer experiences can facilitate the receipt of support [41]. When families engage in more cancer-related discussions, these interactions can be opportunities for social support exchanges [13,33]. For instance, when patients and their family members or caregivers communicate openly about cancer, they have opportunities to provide or exchange emotional support (e.g., listening, validating feelings) [33,42,43,44]. Additionally, perceived social support from family members may help facilitate openness or the discussion of difficult topics [39,41]. For example, people feel more comfortable discussing difficult topics when they feel supported by family members. Furthermore, social support has been linked with better health outcomes, including less burden and depressive symptoms for caregivers [45,46] and less distress, side effects, and lower mortality rates for patients [47,48,49]. Numerous studies have also linked the availability and perceived adequacy of social support with better disease adjustment for patients, caregivers, and family members [50,51,52].

Studies have not examined topic avoidance in hematological cancer caregiving or explored variables (such as openness and support) that may influence avoidance within adult-child–parent relationships. Previous studies have found that openness (i.e., the willingness to communicate about health) [18,19,20] may be an important covariate to consider when assessing topic avoidance as people vary in their willingness to talk about health issues. With these research gaps in mind, we posited the following inquiries:

RQ1: What do caregivers of a parent with hematological cancer report doing after diagnosis that they perceive enhances family communication?

RQ2: What cancer-related topics do caregivers perceive as the most challenging to discuss with their parent, and what topics do they perceive as the most challenging for their parents to discuss with them?

RQ3: Are there differences in the cancer-related topics that are perceived as challenging by the caregiver to discuss versus those they perceive as challenging for their parent?

RQ4: When controlling for caregiver-diagnosed parent openness, is caregiver social support predictive of specific caregiver cancer-related avoidance topics?

RQ5: When controlling for caregiver-parent openness, is caregiver social support predictive of specific parent cancer-related avoidance topics?

## 2. Materials and Methods

### 2.1. Recruitment and Procedure

As part of a larger online survey study focused on developing a supportive intervention for midlife adult-child caregivers of diagnosed parents, we analyzed survey data using both quantitative and qualitative approaches. Upon receiving IRB approval [IRB201902191], caregivers who were part of the Leukemia & Lymphoma Society (LLS) constituency were recruited via email to participate in the survey (this includes caregivers of patients with leukemia, lymphoma, myeloma, myelodysplastic syndromes, and myeloproliferative neoplasms). Recruitment information was also posted in the LLS Community, which is LLS’s private online social network for patients and caregivers. Dissemination began at the height of global quarantine orders during the COVID-19 pandemic from March 2020–June 2020. Recruitment information included a link for interested caregivers to direct them to the survey hosted in Qualtrics, where they first answered screening questions to confirm eligibility. To be eligible, caregivers had to be (a) at least 18 years old, and (b) caring for a living parent, step-parent, or parent-in-law with a blood cancer who was still in treatment or who had completed treatment not more than one year ago. Eligible participants then provided consent and completed the survey, which included both closed and open-ended questions that were integrated throughout the survey. Demographics were obtained at the end of the survey. Individuals received a USD 25 gift card for participation.

### 2.2. RQ1: Item and Analysis

Using the constant comparative approach [53,54] we thematically analyzed responses to one open-ended question (What have you done to enhance communication with your parent and other family members since your parent’s diagnosis?). ATLAS.ti was used to manage the data. We inductively analyzed the responses to identify themes using constant comparative analytical steps: (1) becoming immersed in the data, (2) open (inductive) coding, (3) collapsing codes into themes, and (4) identifying thematic properties (axial coding). Standard thematic saturation criteria of repetition and recurrence were used to identify themes. A qualitative expert (CLF) oversaw the initial analysis conducted by another author (TV) and independently analyzed data associated with each theme. Multiple analytical meetings were held to discuss collapsing codes into themes or thematic properties for codebook development. A final codebook was developed that was then reviewed and approved by a content expert (CLB). To further ensure rigor, an additional coder (MDM) used the codebook to validate the analysis using a closed-coding approach and meetings were held with CLF to collapse thematic properties and confirm saturation at the theme and property levels [55]. Patterns were deemed themes when reported by at least 10% of respondents to the item, with most themes reported by at least 30% of respondents. Quantitative analyses included conducting descriptive statistics, *t*-tests, and regression analyses using SPSS Version 24.

### 2.3. RQs 2–5: Quantitative Measures

In addition to demographic information, several scales were used to measure their openness, topic avoidance, and social support perceptions. All measures for the current study were established scales used in previous studies. Scale reliability was assessed using Cronbach’s alpha.

#### 2.3.1. Caregiver–Parent Cancer Communication Openness

Participants responded to the 4-item Openness to Communicate about Cancer in the Nuclear Family scale [56]. Higher scores indicated more openness. Items formed a reliable scale (*α* = 0.75, *M* = 3.12, *SD* = 0.92).

#### 2.3.2. Caregiver Cancer-Topic Avoidance

Participants responded to the Cancer-Topic Avoidance scale [47], which was slightly modified for parent–child relationships. Caregivers completed the scale twice—once to report personal topic avoidance and a second time to report their perception of the parent’s avoidance. Items were rated on a 5-point scale of (1) “strongly disagree” to (5) “strongly agree”. Higher scores indicated more avoidance. Scale reliability was good for both the caregivers’ report of personal avoidance (*α* = 0.95, *M* = 2.86, *SD* = 0.83) and their parent’s avoidance (*α* = 0.95, *M* = 2.88, *SD* = 0.84).

#### 2.3.3. Social Support

Participants responded to the 8-item Functional Social Support Questionnaire [57]. Items were rated on a 5-point scale of (1) “much less than I would like” to (5) “as much as I would like”. Higher scores indicated more perceived social support. Items formed a reliable scale (*α* = 0.91, *M* = 3.68, *SD* = 1.03).

## 3. Results

### 3.1. Participant Characteristics

In total, 121 adult-child caregivers completed the survey. Of these, 84 answered the open-ended question. See Table 1 for participant demographics.

### 3.2. Qualitative Findings (RQ1): Approaches to Enhancing Family Communication

Responses to the open-ended survey item ranged from 1–169 words, with an average response of 28 words. Caregivers reported six approaches that they perceived enhanced family communication after their parent was diagnosed. Properties of each theme (i.e., approach) are also presented to illustrate how each approach was enacted to enhance family communication. These findings are also summarized in Table 2.

#### 3.2.1. Utilizing Digital Communication Modalities

Caregivers wrote about establishing new modes of family communication via technology (e.g., email, WhatsApp, Zoom). They described how technology promoted better communication by helping them feel and stay connected. At times, new technology was initiated to cope with COVID-19 social distancing orders (e.g., “Lately, we are video chatting with him [father] since we are not allowed to visit.” (41)). These new modalities promoted family connection: “We all purchased Portals (video phones) so Mom can communicate and see her children and two grandkids” (28). Technology also helped to keep information accessible to family. This was particularly important when parents were not home or to keep families informed: “When she is in the hospital, we kept a share[d] google doc of what the drs/nurses said. The My Chart portal has been really helpful in keeping us all in the loop” (39). Caregivers also described how they used technology to maintain or facilitate group communication, which promoted dialogue: “[We have] constant communication via text group so we are all on the same page” (100). Group communication facilitated communal coping by helping families bind together: “We group text information to my siblings to keep them in the loop. ... We have family prayers through texts and phone calls. Honestly that has helped significantly too” (16).

#### 3.2.2. Prioritizing Frequent Communication

Caregivers prioritized frequent communication, which seemed more possible by having new communication modalities. They expressed the need to increase interactions and maintain “constant” contact (“daily”, “hourly”, or “weekly”). Frequent communication helped families maintain contact. Caregivers wrote about dedicating time to check in with their parent and family members, including “scheduled discussions” (26): “[I] make sure that I speak to my mother every day” (63). Frequent communication also helped caregivers attend to caregiving tasks/needs: “We talk daily regarding his condition and what is needed for the day” (41). Another caregiver wrote about her communication with her mother: “I make sure I check on her hourly, whether she’s eating properly or not” (98). Frequent contact could also facilitate relational connection, which included relational communication or communication about topics other than cancer. Caregivers observed an increase in family communication (in-person and over-the-phone) (e.g., “I just talk to my father more. I think we have spoken more in the past three months than the past 30 years” (94)) and linked this with more time talking and engaging in family activities, which promoted togetherness: “I eat more meals with my family so we can all talk and have a distraction of food [from cancer]” (110).

#### 3.2.3. Engaging in Open Communication

Caregivers identified how openness enhanced family communication and was linked with frequent communication. Caregivers had to actively facilitate openness by being the one to broach conversations (e.g., ‘be[ing] proactive about initiating conversation” (20)). Facilitating dialogue included asking questions: “I spend a lot of time at my parents’ house and try to ask lots of open-ended questions” (101). Caregivers sometimes referred to the importance of openness generally (e.g., “Just be open and help” (114)). They also identified how openness was used specifically to promote honesty, particularly with parents (e.g., “Continue to have the conversations. And no secrets! It’s all out there!” (5)). This included openness with other members: “I speak openly with my mother and father and text/call multiple times a day so I do not keep anything from my mother” (95). Openness ensured that everyone could disclose needs and emotions: “We just encourage each other to express our feelings” (83). This included sharing opinions about care and opening dialogue: “I’ve been open with them [about] the pros and cons of every decision I make… I ask them their opinion and [we] respect each other’” (36). Some caregivers acknowledged that openness was a new approach in their family: “My dad actually has grown much more comfortable expressing vulnerability and emotions, which I think has helped drastically” (13).

#### 3.2.4. Establishing Communication Boundaries

Caregivers created boundaries in their communication with family. They were selective about who to interact with, when to interact, and what to interact about, which allowed caregivers to better function in their caregiving role (e.g., “I have chosen only certain family members that we communicate with, and they will usually call the other family members to give information as needed” (1)). Creating boundaries helped to protect parents from distress. When to interact was decided in relation to their parent’s well-being: “[I] try to make sure I don’t discuss important matters with my mom during chemo week or the week after when her mind is not always clear” (15). This included giving parents control about when/what to disclose: “Recently my mom has felt more comfortable letting her siblings know she is ill. In the beginning she didn’t want them to know. She didn’t want the questions and to hear the doom and gloom” (14). Boundaries about who to share information with (or what to share) helped buffer caregivers from distress: “I keep communication to a minimum, try to avoid arguments, don’t try to defend myself because I have to conserve my energy and think about my health” (10).

#### 3.2.5. Being the “Kinkeeper”

Caregivers perceived that their role as “kinkeeper” enhanced family communication. Communication was enhanced because they would share information with family: “I always update all family members on her status” (30). This included using technology to share information and facilitate group communication: “After each appointment I attend, I will follow up with my siblings about what happened and what next steps are. I also created one email account that my sisters and I share” (90). As kinkeepers, caregivers enhanced communication because they would also encourage family involvement. At times this centered on sharing the caregiving load: “I tried to have them get involved with her treatment” (103). Involvement also promoted togetherness: “I’ve also hosted visitations with family where they can come visit, and I take care of her so all they have to do is visit with her” (109).

#### 3.2.6. Enacting Social Support

Caregivers reported that family communication was also enhanced through three types of social support, which seemed related to their “kinkeeper” role. They thought it was important to provide emotional support, including positive communication to facilitate adjustment (e.g., “I decided to concentrate to make Mami feel as happy as she can be. She is 84 years old!” (34)). Caregivers also provided emotional support by listening and being patient: “She’s not herself/in her right mind and may never be and, as a result, may not have the capacity to talk about difficult things or see my perspective as she used to. As a result, I’m trying to be ultra-patient and let mean things she might say roll off my back—to deal with my anxiety and fears privately and just listen to/engage in whatever she wants to talk about” (27).

Caregivers also shared that it was important to offer instrumental support (e.g., going to appointments, performing household tasks, managing medications). A caregiver shared, “We attend all clinic visits and are encouraged to ask our own questions with the healthcare team. We also all attempt to stay on top of her results, upcoming appointments, imaging (side effect management), physical exercise, diet” (6). Caregivers also would give informational support, seeking information from outside sources (e.g., therapy, psycho-educational resources) which was shared with family to enhance communication: “Some of us have sought our own support outside (webinars, counsellors) to deal with the situation on our own outside of the household. This has been helpful as it’s provided communication tools, suggestions” (6).

### 3.3. Quantitative Results (RQ2–5): Exploring Openness and Support

Caregivers identified topics they perceived as the most challenging to discuss with their parent and their perceptions of the most challenging topics for their parents to talk about (RQ2). Caregivers’ perceptions of their own and parent’s challenging topics were similar (see Table 3 and Table 4), with issues related to mortality as the most challenging to discuss, followed by disclosing negative emotions. The challenging topics are arranged in the tables from most challenging to least challenging for adult caregivers to discuss with their parents and caregivers’ perceptions of the most challenging topics for their parents to discuss with others.

We examined differences between adult-child caregivers’ perceptions of cancer-related topics they find challenging to discuss versus those that are challenging for their parents to discuss (RQ3). A related-measures *t*-test revealed that caregivers perceived that their parents engaged in significantly greater topic avoidance regarding burden (M = 2.74; SD = 0.92) versus their own avoidance of the topic of burden (M = 2.57; SD = 0.86) (related-samples *t* = −2.781, *p* < 0.01). Related-measures *t*-tests revealed that caregivers perceived that their parents engaged in significantly greater topic avoidance regarding feelings/emotions (M = 3.08; SD = 0.1.11) versus their own topic avoidance regarding feelings/emotions (M = 2.88; SD = 0.09) (related-samples *t* = −2.486, *p* < 0.05). There was no difference between adult-child caregivers’ perceptions versus those of their parents in terms of topic avoidance concerning the discussion of death (*t* = 1.707, *p* > 0.05) or treatment (*t* = −1.052, *p* > 0.05).

RQ4 assessed the relationships between caregiver-perceived social support and specific cancer-related topics caregivers avoid (controlling for openness). A regression analysis indicated that, when controlling for caregiver–patient openness (M = 11.47; SD = 3.72), increased perceived caregiver social support (M = 29.43; SD = 8.24) was predictive of lower avoidance with regard to discussing cancer treatment (M = 2.39; SD = 0.94); β = −0.22, *t* = −2.780, *p* < 0.01. Moreover, when controlling for caregiver–patient openness (M = 11.47; SD = 3.72), increased perceived caregiver social support (M = 29.43; SD = 8.24) was predictive of lower avoidance with regard to discussing cancer burden (M = 2.55; SD = 0.86); β = −0.23, *t* = −2.938, *p* < 0.01. Finally, when controlling for caregiver–patient openness (M = 11.47; SD = 3.72), increased perceived caregiver social support (M = 29.43; SD = 8.24) was predictive of lower avoidance with regard to discussing feelings related to cancer (M = 2.87; SD = 1.04); β = −0.24, *t* = −2.973, *p* < 0.01.

RQ5 assessed the relationship between caregiver-perceived social support and specific cancer-related topics parents avoid (controlling for openness). A regression analysis indicated that when controlling for caregiver–patient openness (M = 11.52; SD = 3.71), increased perceived caregiver social support (M = 29.57; SD = 8.13) was predictive of lower avoidance with regard to discussing death (M = 3.23; SD = 0.87); β = −0.20, *t* = −2.345, *p* < 0.05. In addition, when controlling for caregiver–patient openness (M = 11.47; SD = 3.72), increased perceived caregiver social support (M = 29.43; SD = 8.24) was predictive of lower avoidance with regard to discussing cancer treatment (M = 2.46; SD = 1.01); β = −0.29, *t* = −3.478, *p* < 0.01. Moreover, when controlling for caregiver–patient openness (M = 11.47; SD = 3.72), increased perceived caregiver social support (M = 29.43; SD = 8.24) was predictive of lower avoidance with regard to discussing cancer burden (M = 2.72; SD = 0.92); β = −0.34, *t* = −4.260, *p* < 0.001. Finally, when controlling for caregiver–patient openness (M = 11.52; SD = 3.71), increased perceived caregiver social support (M = 29.43; SD = 8.24) was predictive of lower avoidance with regard to discussing feelings related to cancer (M = 3.06; SD = 1.13); β = −0.25, *t* = −2.975, *p* < 0.01.

## 4. Discussion

These findings highlight approaches that adult-child caregivers of a parent with hematological cancer use to promote open, supportive communication between a caregiver and parent and with the broader family system. The qualitative findings naturally revealed that blood cancer caregivers characterize openness and support as ways to enhance family communication after diagnosis. The quantitative results extend these findings by identifying how openness and social support intersect, revealing less topic avoidance in more open, supportive caregiver–parent bonds. Collectively, the findings provide direction for tailoring interventions to enhance skills among midlife adult-child caregivers who may struggle with communicating with their diagnosed parent and family members.

The qualitative results indicate that caregivers perceive family communication is enhanced when they communicate openly and frequently. They shared how using technology helped them maintain contact, facilitate connectedness, share information, and encourage involvement. They also described strategies for navigating openness such as practicing honesty, asking open-ended questions, expressing emotions, and setting boundaries. The quantitative results extend this by elucidating contextual information about when openness may be most challenging by identifying cancer-related topics they perceive as the most challenging to discuss. These findings have implications for intervention development in that they identify communication skills caregivers need help with in developing (i.e., openness) as well as caregiving contexts (e.g., talking about mortality or negative emotions) that especially warrant attention in developing open communication skills. Helping families talk openly in general but also about these critical care topics has implications for their quality of life. For instance, families that do not have end-of-life conversations report tension, whereas families that do describe opportunities for relational repair, connection, and a better grieving process [58]. Additionally, disclosing negative emotions is linked with less depression and anxiety and better cancer adjustment for patients and caregivers [59].

When assessing the relationship between social support and topic avoidance, the quantitative results also demonstrate that openness may be an important covariate that contributes to caregivers’ perceptions of feeling supported. The quantitative findings indicated that caregivers’ perceptions of support influenced the degree to which they were open about cancer; those who perceived more support reported less topic avoidance. These findings also provide implications for interventionists aiming to build resources to better support caregivers’ needs. Specifically, our results indicate that interventions that help families become more comfortable with openness and how to broach certain topics are also critical to promote better support the well-being of both patients and caregivers [43,44,45,46,48,49,50,60,61]. Moreover, openness and support can go hand in hand. Families might encounter more opportunities for social support exchanges if they are more willing to communicate [33,41]. When controlling for openness, those who reported less support were more likely to avoid cancer discussions. This held true for specific topics. Those who perceived more support also reported less topic avoidance with regard to treatment, burden, and negative feelings. However, social support did not predict avoidance about the most avoided topic—mortality—which signals a topic that caregivers need additional help with in discussing with their diagnosed aging parents. Discussions about death are perceived as taboo and fraught with emotions and, thus, commonly avoided in all cancer caregiver relationships (e.g., spousal, parent–child) [30,31]. It is likely that adult children and parents may especially struggle with this topic. Adult children described the importance of communication boundaries to protect their parents from distress. They may not only feel protective of their parent’s well-being in their caregiver role, but adult children may perceive it to be inappropriate to initiate certain discussions, such as about mortality, given their role as child in the relationship. At the same time, parents may try to protect their children by not discussing challenging topics, particularly concerns related to their own mortality [25,33]. Diagnosed parents across all ages have described avoiding conversations or withholding information to prioritize their role as a parent and wanting to buffer their children, even in adulthood, from emotional distress [13,25,33].

Technology may help adult-child caregivers communicate more openly with their parents and facilitate their “kinkeeper” [62] role, which is likely new for them or a relational role shift as the adult child in the bond. Studies show how digital communication (e.g., “family-connecting technology”) [63,64] can help kinkeepers keep family members informed and connected. This may be especially important during the COVID-19 pandemic and in the context of hematological cancer as patients experience more isolation. Caregivers described how technology helped them keep families in the loop by sharing information and staying connected. Group chats/texts allowed more family members to have a voice in decision-making with the diagnosed parent and promoted togetherness. Technology may even encourage communal coping, such as seeing cancer as “our” problem and pooling resources to cope—a supportive approach associated with better health outcomes [65,66]. Communal coping opportunities via technology may facilitate social support exchanges and alleviate the burden for diagnosed parents and their adult children, while at the same time helping adult children shift into their role as the caregiver of a parent [67,68]. It may also further enhance the parent’s comfort in being more dependent on their child for assistance. In interventions that focus on developing open and supportive communication skills, it would be helpful to include suggestions for using technology and to promote the adult-child caregiver’s and their diagnosed parent’s comfort with digital communication.

Scholars and practitioners have argued for the need for policy changes to reduce both cancer patients’ and their caregivers’ suffering [69], particularly since the COVID-19 pandemic as this global health crisis exacerbated the challenges and load expected of family caregivers [70]. Findings provide further evidence for the need for policy changes that support “frontline family caregivers” such as adult children of parents living with a blood cancer who have become more reliant on loved ones not only for care, but also for protection from COVID-19 risk [70].

### Limitations

Caregivers identified mostly as white and female and represent those who have engaged with an advocacy organization. Daughters are more likely to fulfill caregiving and kinkeeping roles, which informs the approaches that they described (e.g., setting boundaries, sharing information as the kinkeeper). Although this study sought both parents and parent-in law bonds, the sample represented parent—child bonds. Future studies could parse out distinctions in experiences with in-law parent–child bonds. Additionally, given that caregiving expectations vary by culture and that communication is also distinct across cultures and gender, future studies should use purposive sampling to examine challenges with openness and avoidance in diverse family cultures as well as male family caregivers’ experiences (e.g., sons) to further understand blood cancer caregivers’ needs. Additionally, dyadic data with both relational partners would more fully represent the challenges with open communication. Our sample was also recruited using an incentive which may have motivated caregivers to decide to participate.

## 5. Conclusions

These findings offer directions for an intervention focused on family communication skill development for adult-child caregivers of a parent diagnosed with a blood cancer. Caregivers can take the lead in facilitating openness and support. Communication skills training should address caregiving topics found to be particularly challenging and model how to broach them. Support staff at the site of care can help patients to initiate and navigate conversations about those topics.

## Figures and Tables

**Table 1:** Adult-child Caregiver Demographics (*N* = 84).

Characteristic	*N* = 84
Participant Age	*M* = 44.82; *SD* = 11.73
Parent’s Age	*M* = 72.45; *SD* = 11.24
Caregiver Sex	Female (*n* = 69; 82.1%) Male *(n* = 15; 17.9%)
Relationship to Parent	Daughter, Stepdaughter, or Daughter-in-law (*n* = 69; 82.1%) Son, Stepson, or Son-in-law (*n* = 15, 17.9%). Only *n* = 4 (0.04%) participants indicated caring for an in-law
Number of Children	*M* = 1.96, *SD* = 1.26
Number of Children ≤18	*M* = 0.87, *SD* = 1.01
Race	Caucasian (n = 65; 77.4%) Asian (n = 9; 10.7%) Black/African American (n = 9; 10.7%) American Indian (n = 1; 1.2%) Hawaiian/Pacific Islander (n = 1; 1.2%)
Ethnicity	Hispanic (N = 10, 11.9%) Not Hispanic (N = 74, 88.1%)
Education (highest level/ degree completed)	High school graduate/GED (n = 7; 8.3%) Some college (n = 7; 8.3%) 2-year degree (n = 7; 8.3%) 4-year degree (n = 27; 32.1%) Master’s degree (n = 26; 31.0%) Professional degree (n = 4; 4.9%) Doctoral degree (n = 6; 7.1%)
Employment Status	Employed full time (*n* = 52; 61.9%) Employed part time (*n* = 6; 7.1%) Self-employed (*n* = 6; 7.1%) Not employed (*n* = 12; 14.3%) Retired (*n* = 8; 9.5%)
Relationship Status	Married/Domestic partner (n = 46; 54.8%) Divorced (n = 11; 13.1%) Single/Never married (n = 23; 27.4%) Widowed (n = 3; 3.6%) Separated (n = 1; 1.2%)

**Table 2:** Approaches to Enhancing Family Communication.

Themes and Subthemes	Illustrative Quotes
1. Utilizing Digital Communication Modalities	
to feel and stay connected	“We all purchased Portals (video phones) so Mom can communicate and see her children and two grandkids.”
to keep information accessible	“When she is in the hospital, we kept a share[d] google doc of what the drs/nurses said. The My Chart portal has been really helpful in keeping us all in the loop.”
to maintain or facilitate group communication	“We group text information to my siblings to keep them in the loop. … We have family prayers through texts and phone calls. Honestly that has helped significantly too.”
2. Prioritizing Frequent Communication	
to maintain contact	“[I] make sure that I speak to my mother every day.”
to attend to caregiving tasks/needs	“I make sure I check on her hourly, whether she’s eating properly or not.”
to facilitate relational connection	“I eat more meals with my family so we can all talk and have a distraction of food [from cancer].”
3. Engaging in Open Communication	
to actively facilitate openness	“I spend a lot of time at my parents’ house and try to ask lots of open-ended questions.”
to promote honesty	“Continue to have the conversations. And no secrets! It’s all out there!”
to disclose needs and emotions	“My dad actually has grown much more comfortable expressing vulnerability and emotions, which I think has helped drastically.”
4. Establishing Communication Boundaries	
to function in their caregiving role	“I have chosen only certain family members that we communicate with, and they will usually call the other family members to give information as needed.”
to protect parents from distress	“Recently my mom has felt more comfortable letting her siblings know she is ill. In the beginning she didn’t want them to know. She didn’t want the questions and to hear the doom and gloom.”
to buffer caregivers from distress	“I keep communication to a minimum, try to avoid arguments, don’t try to defend myself because I have to conserve my energy and think about my health.”
5. Being the “Kinkeeper”	
to share information with family	“After each appointment I attend, I will follow up with my siblings about what happened and what next steps are. I also created one email account that my sisters and I share.”
to encourage family involvement	“I’ve also hosted visitations with family where they can come visit, and I take care of her so all they have to do is visit with her.”
6. Enacting Social Support	
to provide emotional support	“I’m trying to be ultra-patient … [to] listen to/engage in whatever she wants to talk about.”
to offer instrumental support	“We attend all clinic visits … and attempt to stay on top of her results, upcoming appointments, imaging (side effect management), physical exercise, diet.”
to give informational support	“[We] sought our own support outside (webinars, counsellors) … This has been helpful as it’s provided communication tools, suggestions.”

**Table 3:** Most challenging topics for adult-child caregivers to discuss with parents.

Topic	Mean	*SD*
Death	3.38	1.01
Feelings/Emotions	2.88	1.03
Burden	2.58	0.86
Treatment	2.40	0.94

**Table 4:** Adult-child caregiver perceptions of the most challenging topics for parents to discuss.

Topic	Mean	*SD*
Death	3.25	0.86
Feelings/Emotions	3.08	1.11
Burden	2.74	0.92
Treatment	2.49	1.02

## Data Availability

The datasets generated during and/or analyzed during the current study are available from the corresponding author upon reasonable request.
